# The Microscope in Medicine

**Published:** 1891-11

**Authors:** B. F. Church

**Affiliations:** Terrell, Texas, Assistant Physician Terrell Hospital for the Insane


For Daniel's Texas Medical Journal.

### THE MICROSCOPE IN MEDICINE.

B. F. CHURCH, M. D., TERRELL, TEXAS,
Assistant Physician Terrell Hospital for the Insane.

Read before the Terrell Medical Society, August 2, 1891.

OF INESTIMABLE value to other sciences the microscope has deservedly been called the leading genius of the science of medicine. It is generally believed that the first compound microscope was discovered in 1590. It was then, and for many years a very imperfect instrument, coloring, and distorting objects observed, and was principally used for a toy.

As history shows in many instances, this time was not without one who lived in advance of his time. Athanasius Kircher, after observing and studying the life history of some of the larger micro-organisms—notwithstanding he could not see many that we know are associated with certain diseases—attempted to demonstrate the germ theory of disease. Others joined him in his belief, but as their theory could not be substantiated it fell into obscurity.

Without the microscope, our present knowledge of anatomy and physiology would be impossible.

The next important use to the physician of this valuable instrument was in the study of pathological anatomy. The foundation of this study as well as that of biology was begun during the seventeenth century. As a natural result of the evolution of the means for wider researches; created mainly by improvements in the microscope, and methods of observation, pathology, at a later day, was scientifically studied.

Now, nearly three centuries after the discovery of the microscope, it enabled those in our day to make such stupendous advances in the science of medicine as to claim the attention of the whole thinking world, transformed empirical blunders of our forefathers into reasonable certainties and almost revolutionized the practice of medicine (in its broadest sense) and hygiene; this is the science of bacteriology. Now, as it is, the study of bacteriology has placed the practice of medicine nearer to an exact science than all other investigations combined.

It is not in the province of this paper to review all the proofs of contagium vivum; Koch's researches, principally, elevated this theory to demonstrable and established fact.

Commensurate with our enthusiasm upon an insight into this hidden world, we are in danger of failure to establish the exact relationship of the bacterium to the disease in question.

It is not sufficient to simply know how to recognize and artificially cultivate bacteria; associated with certain diseases, they may, or may not be their causative agents.

The action of the body cell in presence of disease germ also requires special consideration.

Koch's proof of the existence of a pathological micro-organism was as follows:

1. The micro-organism must be found in the blood, lymph, or diseased tissue of man or animal suffering from or dead of the disease.

2. The micro-organisms must be isolated from the blood, lymph, or tissues, and cultivated in suitable media, i. e., outside the animal body. These pure cultivations must be carried on through successive generation of the organisms.

3. A pure cultivation thus obtained must, when introduced into the body of a healthy animal, produce the disease in question.

4. Lastly, in the inoculated animal the same micro-organism must be found.

In this connection, Crookshank* says: "The determination of the true pathogenic microbe is beset with many fallacies. In many diseases bacteria have been regarded as the actual contagia, until a searching inquiry by other investigations has shown that the evidence was most unsatisfactory, or entirely miseading. For example in diseases with lesions of the external, or internal linings of the body, extraneous micro-organisms may get into the circulation and be swept into the internal organs which are opposed to their existence, or they may gain the upper hand and set up destructive processes. Such organism,

*Crookshank's Manual of Bacteriology, 1891.

when found in association with these diseases, may be discovered in the blood and internal organs; and though accidental epiphytes, often associated with septic complication, they may only too readily be accepted by the enthusiast as the actual contagium of the disease in question.

"It is only when such fallacies are exposed that we are brought face to face with the fact that the nature of the contagium in many diseases, such as hydrophobia, variola, vaccinia, scarlet fever and measles, is at the present day undetermined. Such cases invite further research; but these fallacies must be borne in mind, or the application of bacteriology to the elucidation of the germ theory of disease will not only be misleading but tend to retard rather than to assist the progress of science."

While the busy practitioner has no time for elaborate researches in bacteriology, he stands in his own light when he neglects the urgent demands which arise almost daily for practical microscopy.

There is no department of medicine in which its value is not manifest. As a diagnostic agent the microscope is indispensable, for with it the diagnosis of many diseased based upon imperfect clinical data may be unerringly confirmed. Its aid is probably more often demanded in urinology diagnosis, and differential diagnosis of genito-urinary diseases, than in any other branch. It is not uncommon to find cases whose urine is loaded with albumen, yet they suffer no inconvenience. On the other hand the most delicate chemical tests may fail to detect albumen in the urine and the patient be in a precarious condition from Bright's,—one of the many diseases which can only be diagnosed by the aid of the microscope.

The fact that the microscope affords *the only means of accurately determining urinary sediment* is better appreciated when we remember the great importance of this excretion. Being not only a complete index of the condition of the urinary organs, but a fair indication of that of many of the more important excreting glands in the body, and the nervous system from a social as well as a medico-legal standpoint, it is sometimes very important to differentially diagnose gonorrhœa and non-specific blennorrhagia.

The presence, or absence, of gonococci tells the tale. Occasionally it is of great moment to detect the source, whether from male or female of a given specimen of urine. The writer is familiar with cases and doubtless others have had the same experience, where the wife attempts to palm off her urine for that of her husband; the discovery of several epithelial cells exposing the imposition.

Bacteriological investigation aided by the microscope has discovered and thoroughly identified the specific *cause* of many of the wide spread and terrible, human diseases such as consumption, typhoid fever, Asiatic cholera, diphtheria, etc., which not only points out to the physician a perfect line of prophylactic treatment but suggests new remedies for their cure, and enables him to concentrate all of his therapeutic agents in the direction of their greatest usefulness.

The nature and mode of communication of yellow fever, scarlet fever, measles, small-pox, etc., strongly indicate that they too are bacterial diseases, but the specific organisms causing them have not been identified. The latest confirmation of bacterial origin of one of the dread diseases is that of diphtheria. The identity of this disease and membranous croup, is no longer a mooted question. Considerable interest has recently been manifested regarding the supposed discovery of the bacterium of malaria. Minute micro-organisms are found in the blood corpuscles and free in the serum, during certain stages of this protean disease. They are not bacteria, but belong to a low form of life called protozoa and we know not whether they are the cause or result of the disease. They may be accidental—impoverishment of blood from some other cause furnishing a proper nidus for their growth and development. At any rate reliable observations so far has failed to definitely mark out the relation of this hæmatozoon to malaria.

To Dr. J. W. McLaughlin, of this State, is due the credit of valuable researches in the causation of dengue. He was the first to isolate from the blood of dengue patients a micrococcus which is supposed to be the infecting agent. No one can surmise the ultimate results to accrue from the impetus given to microscopy by the undreamed of revelations of the last few years.

The diameter of the smallest object now visible with the microscope is one sixteen thousandth of a millimeter. It is just reported though that by improvement in the manufacture of glass and grinding of lenses an object only *one eight millionth* of a millimeter in diameter can be seen.

